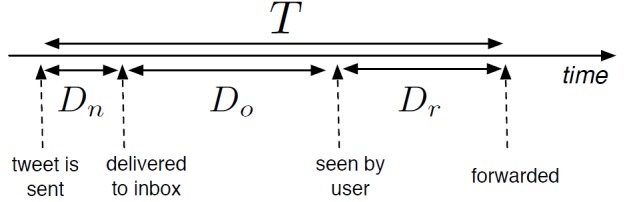# Correction: Lognormal Infection Times of Online Information Spread

**DOI:** 10.1371/annotation/eb5dda55-3e85-4556-90ac-b7c54acfe306

**Published:** 2013-11-13

**Authors:** Christian Doerr, Norbert Blenn, Piet Van Mieghem

Figure 2 has been updated for better readability. Please see the correct Figure 2 here: 

**Figure:**